# A study of the evolutionary game of carbon offset involving tourism stakeholders under incentive and constraint mechanisms

**DOI:** 10.1038/s41598-024-65964-8

**Published:** 2024-06-28

**Authors:** Qianna Li, Changjiang Xiong, Juan Yao

**Affiliations:** 1 College of Economics and Management, Neijiang Normal University, Neijiang, 641000 China; 2 Institute of Finance and Economics, Shanghai University of Finance and Economics, Shanghai, 200433 China; 3 College of Economics and Management, Xinjiang Agricultural University, Urumqi, 830052 China

**Keywords:** Tourism carbon offset, Evolutionary game, Local government, Tourism firms, Tourists, Climate-change adaptation, Climate-change mitigation, Climate-change policy, Environmental economics, Psychology and behaviour, Socioeconomic scenarios

## Abstract

Tourism carbon offsetting is a crucial pathway to achieving peak carbon and carbon neutrality in the tourism industry. Accurately grasping the collaborative evolutionary mechanisms among local governments, tourism enterprises, and tourists is key to promoting the implementation of tourism carbon offsetting. By constructing an evolutionary game model involving local governments, tourism enterprises, and tourists in carbon offsetting, this study uses MATLAB to simulate the evolutionary stable strategies under various conditions. Additionally, it dynamically simulates the collaborative strategies of the three parties under the influence of local government incentive and constraint mechanisms. The results indicate that under strong governmental constraint mechanisms, there is a promotion of active participation in carbon offsetting by local governments, tourism enterprises, and tourists. Incentive policies at certain levels also play a positive guiding role. As incentives increase, local subsidies and intervention costs also rise, leading to an evolution towards less enthusiastic participation among the three parties. Appropriately balanced government incentives and penalties are beneficial in achieving an equilibrium of benefits among multiple stakeholders involved in carbon offsetting, thus helping to attain carbon neutrality goals.

## Literature review

Tourism heavily relies on natural resources and the ecological environment, making its impact on climate change significant and increasingly concerning^1^. According to the World Tourism Organization and the United Nations Environment Programme (UNWTO & UNE), the tourism sector’s carbon emissions are projected to increase by 152% by 2035, contributing 188% to global warming^2^. In 2021, the UNWTO issued *The Glasgow Declaration*, committing to halving global tourism CO_2_ emissions by 2030 and achieving carbon neutrality by 2050^3^. The question of how to put into action to achieve the global carbon peaking carbon neutral target has become a focal point. Carbon offset has been widely noticed as an effective mechanism to remove carbon dioxide from the atmosphere and achieve carbon reduction targets. In the tourism sector, carbon emitters offset their own carbon emissions in an economic or non-economic way^4^. For example, they pay to comply with the emission cap^5^, or reduce carbon emissions by planting trees and low carbon behaviors^6^. Carbon offset involves a number of stakeholders in the implementation process, and only when the stakeholders reach a consensus on their collective behaviors can they achieve the goal of carbon reduction. It is evident that carbon offset in the tourism industry is an effective institutional arrangement for achieving carbon reduction in tourist destinations and carbon neutrality in the tourism industry.

Currently, there are few studies utilizing evolutionary game models to explore cooperative mechanisms among stakeholders involved in carbon offsetting. Therefore, with the premise of bounded rationality and maximization of self-interest for stakeholders’ strategic choices, this paper constructs an evolutionary game model involving local governments, tourism enterprises, and tourists. It analyzes the equilibrium conditions of the tripartite game and simulates the system’s evolutionary stable strategies under different circumstances. These insights will provide valuable references and enlightenment for the formulation and implementation of tourism carbon offset policies.

### Tourism carbon offset

The term “carbon offset” was introduced in the *Kyoto Protocol* in 1997 as a cooperative mechanism to help countries reduce greenhouse gas emissions^7^, offsetting domestic emissions through the purchase of carbon credits in the carbon market^8^. As a way to “neutralize” emissions, it has received extensive attention from scholars, and the academic community has mainly focused on forest carbon offset^9,10^, agricultural carbon offset^11^, fishery carbon offset^12^, watershed carbon offset^13^, and tourism carbon offset^14–16^.

Scholars have explored the efficacy and feasibility of implementing tourism carbon offset policies, with diverging perspectives. Scott et al. argued against the implementation of such policies, citing cost and reputational risks^17^. It is clearly unfair to limit the development opportunities of developing countries to maintain the interests of the developed groups^18^. On the other hand, an objective perception of the environment takes an opposing view, scholars have emphasized the significant environmental impact of air travel and the need for carbon offset policies. arguing that tourism has a significant impact on climate change and may continue to increase^4^. In particular, air travel contributes to 8% of the global carbon footprint, leading to a significant environmental impact and the need for carbon offset policies. The question of responsibility for carbon offset arises: most tourists believe that governments and airlines should take responsibility for the climate change caused by tourism^19^.

Meanwhile, Gössling proposed a carbon offset model, whereby emissions from one sector can be offset by reductions in another, such as afforestation or renewable energy^18^, offering an acceptable solution for travelers and the tourism industry, contributing to low carbon awareness, and providing broad social benefits^20^. With the increasing implementation of carbon offset models, more countries and organizations recognize the significant role that carbon offset can play in coordinating regional ecological conservation and promoting environmentally friendly economic development^21^. Particularly in the context of the ‘two-carbon’ goal, reconciling environmental issues arising from human tourism activities with the climate policy objectives of the international community has become a priority, with emphasis placed on low carbon and environmental protection in different areas^22^. Research has focused on sectors such as aviation, hotels, and accommodations^23,24^, with the aviation sector in particular receiving attention in academic circles for identifying ways to reduce greenhouse gas emissions without negatively affecting tourism activity. Measures being discussed include air passenger taxes, emission trading schemes, emissions taxes, fuel taxes or VAT, technological changes (improved fuel efficiency, alternative fuels), and structural changes (restructuring of public transport, capacity management)^25,26^.

Research on tourism carbon offset decision-making behavior mainly focuses on stakeholder subjects such as governments, residents, tourists, and tourism enterprises. Sautter classifies tourism stakeholders into government departments, tourists, residents, employees, local merchants, competitors, activist groups, and so on^27^. while Sward adds pressure groups, public interest organizations, experts, and the media^28^. Some scholars also distinguish between core and peripheral interest subjects according to different spatial regions^29^. For instance, research on rural tourism focuses on the relationship between communities and scenic spots, tourism enterprises, and tourists, whereas tourist attractions and resorts emphasize the relationship between scenic spots and government, travel agencies, enterprises, and tourists. Eco-regions, such as forest parks and heritage sites, concentrate on the relationship between local governments and operating companies, scenic spot management agencies, practitioners, tourists, and residents. These relationships often face contradictions and conflicts due to economic needs, different values, and inadequate management systems^30–32^. Therefore, effectively coordinating the relationship between different stakeholders and maximizing their participation in carbon offset is the key and challenging aspect of carbon emission reduction governance.

### Low-carbon tourism and the evolutionary game of carbon reduction

Traditional game theory assumes that decision-makers are perfectly rational and that information between decision-makers is not fully transparent, which is difficult to achieve in real life^33^. In contrast, evolutionary games consider decision makers to be finitely rational and can dynamically study the strategies of multiple actors, analyzing the effects of different factors on the change process of decision makers’ decisions^34^. As a result, scholars have constructed evolutionary game models to analyze the interests between carbon pollution stakeholders in different countries or regions, with a focus on low-carbon tourism and tourism carbon reduction^35^. Low-carbon tourism is a new way to reduce carbon emissions^36^, and it involves tourists, government departments, community residents, and tourism enterprises. Tourism enterprises are the implementers of low-carbon tourism, with the use of renewable energy and energy conservation as their top priority. Government departments play a coordinating and guiding role, promoting environmental awareness through improved carbon sink mechanisms, monitoring mechanisms, and management bodies^37^. Local communities are the participants and beneficiaries, while tourists are the experiencers of low-carbon tourism^38^. The respective positions of stakeholders and their interrelationships have become the content of low-carbon tourism research. It is found that the decisions of enterprises and tourism consumers are influenced by each other. The government can effectively increase the enthusiasm of tourism consumers and enterprises to participate in low-carbon tourism by setting reasonable subsidies and penalties for tourism consumers and enterprises^39^.

Carbon emission reduction is essential for achieving peak and neutral carbon targets, and quantification is necessary for the development of carbon offset schemes. In public goods games like carbon reduction, the use of incentives and disincentives to solve the cooperation dilemma of stakeholders has garnered scholarly attention^40–43^. Using the evolutionary game model, it has been inferred that carbon emission reward and penalty mechanisms are effective in promoting low-carbon development among enterprises. If the penalty for tourism enterprises exceeding carbon emissions is too low, and the cost of local government supervision is too high, the government may abandon regulation^44^. The intervention of the central government plays a non-negligible role in the implementation of carbon offset policies between regions, and the implementation cost and intensity of incentive and constraint mechanisms will affect the time required for the implementation of carbon offset policies in the region^21^. The optimized dynamic punishment incentive mechanism can effectively inhibit the fluctuation of interest selection strategy^45^, and government subsidies can improve the total social welfare, but the lower limit of subsidies should not be too high^46^. These studies have shown that properly designed incentives and constraints can effectively promote low-carbon development and carbon-offsetting policies. These findings can help policymakers develop effective carbon reduction strategies and facilitate the transition to a low-carbon economy.

In summary, current research has focused on the game between the parties involved in low-carbon tourism behavior and tourism carbon reduction behavior. However, few studies use evolutionary game models to explore the cooperative mechanisms of stakeholder carbon offsets. Therefore, it is necessary to study the cooperative evolutionary strategies of stakeholders’ participation in tourism carbon offset and to identify solutions for implementing tourism carbon offset policies. The structure of this paper is as follows: Section One reviews the research progress on the involvement of stakeholders in low-carbon tourism, tourism carbon reduction, and the carbon offset game. Section Two constructs a tripartite evolutionary game model involving local governments, tourists, and tourism enterprises, applies the Lyapunov criterion to derive the system’s evolutionarily stable strategies under various conditions, and analyzes the stability of the tripartite evolutionary equilibrium points. Section Three simulates the stable equilibrium strategies for the three parties participating in carbon offsets under government incentives and constraints. Section Four summarizes the research findings and proposes relevant policy recommendations. The marginal contributions of this study include the following two points: First, against the backdrop where the tourism industry will become the main battleground for energy conservation and emission reduction globally, it explores cooperative strategies among stakeholders involved in tourism carbon offset activities, including tourists, government agencies, and tourism enterprises. The strategies they adopt will directly impact the effectiveness of energy conservation and emission reduction in the tourism industry, thereby enriching the theory of carbon neutrality in tourism. Second, it defines the effective range of constraints and incentives for government implementation of tourism carbon policies, driving the system towards different stable states. This has significant reference value for managers to scientifically and reasonably formulate tourism carbon offset mechanisms.

## Tripartite evolutionary game modeling

### Basic assumptions

#### The hypothesis of tourism carbon offset participants

This paper simplifies the complex tourism carbon offset relationship system, based on the criteria of the highest carbon emission impact and carbon governance contribution^47^. Three representative subjects of tourists, tourism enterprises and local governments are selected to form a multivariate collaborative governance game model^48^. Under the model of tourism carbon offsetting, local governments, tourism enterprises and tourists are limited rational economic agents, all aiming to realize their own maximum interests^39^. Local governments give primary consideration to the realization of overall and long-term interests, as well as prioritizing the image of the government. On the other hand, tourism enterprises prioritize short-term and vested interests and strive to maximize profits or minimize costs. Similarly, tourists pursue short-term and vested experiences of quality services, and maximize utility or minimize consumption costs as a code of conduct^39^. As limited rational subjects, the strategic behavioral choices of local governments, tourism enterprises and tourists tend to be optimal strategies.

#### The hypothesis of strategy types in tourism carbon offset

It is hypothesized that following the implementation of tourism carbon offset practices by the central government, local governments, tourism enterprises, and tourists can adopt different strategies. Specifically, local governments can choose to enforce strong (x_1_) or weak (x_2_) enforcement strategies. Similarly, Tourism enterprises can choose between proactive response (y_1_) and passive response (y_2_), while tourists have the options of participating (z_1_) or not participating (z_2_).

It is important to note that x, y, and z are all within the range of 0 to 1, indicating that the stakeholders’ strategies are not absolute but are relative to their interests and circumstances. By adopting these strategies, local governments, tourism enterprises, and tourists aim to maximize their benefits while minimizing costs^49^.

#### Payment function assumptions

1. Local governments: When the local government chooses strong enforcement, it will reward tourism enterprises that actively respond to carbon offsets (W_1_) and tourists that actively participate in carbon offsets (W_2_)^39^. and at the same time, the local government needs to pay for the enforcement cost of the regulatory process (C_1_)^50^. Local governments can reap hidden environmental benefits (R_1_)^51^ and image benefits (V)^52^ from carbon offsets. When the government plays the function of environmental governance, there is the phenomenon of “the law does not punish numerous offenders”^53^, i.e., due to the existence of penalty costs, managers tend to impose smaller penalties or difficult to impose penalties on group violations, while the penalties for individual violations tend to be larger, and therefore penalize the tourism enterprises that are passive (F_1_)^54^. Meanwhile, when governments choose to implement negatively, if tourists choose a non-participation strategy and tourism enterprises choose a negative response strategy, the local government does not need to pay the enforcement cost, but the local government may suffer a loss of government reputation and credibility due to failure to fulfill the tasks assigned by the central government (I), and bear the environmental risk cost (L)^54^.
2. Tourism enterprises: When a tourism company chooses a positive coping strategy, it will obtain a low carbon benefit (R_3_)^55^, and needs to pay the operating cost (C_3_), but at the same time, it can obtain the corresponding carbon offsetting incentive (W_1_)^39^. When a tourism enterprise chooses a negative coping strategy, it will obtain a gain (R_2_) (R_2_ < R_3_), in the short term, the production enterprise can obtain a considerable gain at the expense of the government and the public’s environmental interests^56^, but in the long term, when a tourism enterprise chooses a negative coping strategy, it will provide tourism products and services that do not comply with the national dual-carbon target policy orientation and market demand, and then its gain will decline. In addition, the enterprises need to pay the common operating costs (C_2_) (C_3_ > C_2_)^50^.
3. Tourists: When tourists choose to actively participate in carbon offsetting, they pay the corresponding participation cost (C_4_) and obtain carbon offsetting incentives from the local government (W_2_)^39^. Tourists pursue the experience utility of low-carbon tourism in tourism consumption activities, and obtain the environmental utility (U_1_)^57^. Tourists choose not to participate in the strategy, pay the corresponding tourism cost (C_5_) (C_5_ < C_4_), and obtain the environmental utility as (U_2_) (U_1_ > U_2_)^58^.

The payoff matrix of the tripartite evolutionary game is shown in Table 1, which illustrates the possible outcomes and payoffs for each stakeholder in the tourism carbon offset process. These payoff functions provide insights into the strategic choices made by stakeholders and help understand the dynamics of carbon offset in the tourism industry.

**Table 1:** Payoff matrix of the evolutionary game between local governments, tourism enterprises, and tourists.

Strategy portfolio	Local government revenue	Tourism enterprises benefits	Tourists benefits
(Strong implementation, active response, engagement) (*x*_1_, *y*_1_, *z*_1_)	−*C*_1_−*W*_1_−*W*_2_ + *R*_1_ + *V*	−*C*_3_ + *W*_1_ + *R*_3_	−*C*_4_ + *W*_2_ + *U*_1_
(Strong implementation, passive response, engagement) (*x*_1_, *y*_2_, *z*_1_)	−*C*_1_−*W*_2_−*I*−*L* + *F*_1_	−*C*_2_−*F*_1_ + *R*_2_	−*C*_4_ + *W*_2_ + *U*_1_
(Strong implementation, active response, non-participation) (*x*_1_, *y*_1_, *z*_2_)	−*C*_1_−*I*−*L*−*W*_1_	−*C*_3_ + *W*_1_ + *R*_3_	−*C*_5_ + *U*_2_
(Strong implementation, passive response, non-engagement) (*x*_1_, *y*_2_, *z*_2_)	−*C*_1_−*I*−*L* + *F*_1_	−*C*_2_−*F*_1_ + *R*_2_	−*C*_5_ + *U*_2_
(Weak implementation, active response, engagement) (*x*_2_, *y*_1_, *z*_1_)	*R*_1_	−*C*_3_ + *R*_3_	−*C*_4_ + *U*_1_
(Weak implementation, passive response, engagement) (*x*_2_, *y*_2_, *z*_1_)	−*I*−*L*	−*C*_2_ + *R*_2_	−*C*_4_ + *U*_1_
(Weak implementation, active response, non-participation) (*x*_2_, *y*_1_, *z*_2_)	−*I*−*L*	−*C*_3_ + *R*_3_	−*C*_5_ + *U*_2_
(Weak implementation, passive response, non-engagement) (*x*_2_, *y*_2_, *z*_2_)	−*I*−*L*	−*C*_2_ + *R*_2_	−*C*_5_ + *U*_2_

### Game model construction

#### Evolutionary game modeling of local governments

The expected benefits $E_{11}$ and $E_{12}$ for local governments choosing strong and weak enforcement, and the average benefits $\overline{{E_{1} }}$ respectively are:

$$  \begin{gathered}   E_{{11}}  =  - C_{1}  - yW_{1}  - zW_{2}  + yzR_{1}  + (1 - y)F_{1}  - (1 - yz)(I + L) \hfill \\   E_{{12}}  = yzR_{1}  + (1 - y)z( - I - L) + (1 - z)( - I - L) = yzR_{1}  + (1 - yz)( - I - L) \hfill \\   \overline{{E_{1} }}  = xE_{{11}}  + \left( {1 - x} \right)E_{{12}}  \hfill \\  \end{gathered}   $$

From this, the first-order derivatives of the replicator dynamics equations for local government $F(x)$, $x$ can be obtained $\frac{dF(x)}{{dx}}$.

$$ {\text{F}}\left( x \right) = \frac{{{d}x}}{{{d}t}} = x\left( {E_{11} - \overline{{E_{1} }} } \right) = x\left( {1 - x} \right)\left[ { - C_{1} - yW_{1} - zW_{2} + \left( {1 - y} \right)F_{1} + {\text{yzV}}} \right] $$

$$ \frac{dF(x)}{{dx}} = (1 - 2x)\left[ { - C_{1} - yW_{1} - zW_{2} + \left( {1 - y} \right)F_{1} } \right] $$

At the same time, making $G\left( y \right) = - C_{1} - yW_{1} - zW_{2} + \left( {1 - y} \right)F_{1}$,

Seeking.$$G\left( {\text{y}} \right){\prime} = - W_{1} - F_{1} + {\text{zV}}$$,

According to stability theory, for local governments, the process of behavioral strategy adjustment tends to a steady state when and only when $F(x) = 0$ and $\frac{dF(x)}{{dx}} < 0$ holds simultaneously.

According to the above assumptions, local governments are mainly pursuing overall and long-term interests, and the image gains obtained by local governments choosing strong enforcement are objective. Assuming that tourism enterprises opt for a proactive response and tourists decide to participate, let us hypothesize that $- W_{1} - F_{1} + zV > 0$. It follows that $G(y)$ is a monotonically increasing function, yielding the solution:

$$ y = \frac{{F_{1} - C_{1} - zW_{2} }}{{W_{1} + F_{1} - zV}} $$

Discussed in three scenarios:

I. The $y = \frac{{F_{1} - C_{1} - zW_{2} }}{{W_{1} + F_{1} - zV}}$, $G(y) = 0$, then $F(x) = 0$, $x$ take any number, are stable states.
II. The $y > \frac{{F_{1} - C_{1} - zW_{2} }}{{W_{1} + F_{1} - zV}}$, $G(y) < 0$, then $\frac{dF(x)}{{dx}}\left| {_{x = 0} } \right. < 0$, $\frac{dF(x)}{{dx}}\left| {_{x = 1} } \right. > 0$,$x = 0$ are evolutionary stability points and the local government’s strategy choice is weak enforcement.
III. The $y < \frac{{F_{1} - C_{1} - zW_{2} }}{{W_{1} + F_{1} - zV}}$, $G(y) > 0$, then $\frac{dF(x)}{{dx}}\left| {_{x = 0} } \right. > 0$, $\frac{dF(x)}{{dx}}\left| {_{x = 1} } \right. < 0$, $x = 1$ are evolutionary stability points and the local government’s strategy choice is strong enforcement.

Based on the above analysis, the following proposition is obtained.

##### Proposition 1

The behavioral strategy of local governments evolves towards weak enforcement as they offer greater incentives to tourism enterprises and tourists.

##### Proof

with all other parameters held constant, will make $y > \frac{{F_{1} - C_{1} - zW_{2} }}{{W_{1} + F_{1} - zV}}$, as $W_{1}$ and $W_{2}$ increase, eventually stabilize at $x = {0}$, i.e. the local government’s strategic behavior is chosen to be weakly enforced. As the incentives offered by local governments to tourism enterprises and tourists increase, there is a higher likelihood that tourism enterprises will respond positively and tourists will participate in carbon offset. However, as incentives continue to grow and local governments become financially overburdened, they may have a higher expectation for active participation from tourism enterprises and tourists in the tourism industry’s carbon offset process, particularly in the context of weak enforcement measures.

##### Proposition 2

The behavioral strategy of local governments evolves towards weak enforcement when the cost of implementing carbon offsets for tourism is higher.

##### Proof

with other parameters unchanged, will make $y > \frac{{F_{1} - C_{1} - zW_{2} }}{{W_{1} + F_{1} - zV}}$, as $C_{1}$ increases, will eventually stabilize at $x = {0}$, i.e. the strategic behavior of the local government is chosen to be weakly implemented. The process of implementing tourism carbon offset, including planning, policy design, and supervision, adds to the governing costs of local governments and may result in lost opportunities for development. As a limited rational economic entity, when local governments have low expectations for tourism carbon offset, they may choose a weak implementation strategy. This highlights the need for increased efforts to guide and encourage local governments to participate in carbon offset initiatives, while also considering the economic costs and benefits involved.

##### Proposition 3

The behavioral strategy of local governments evolves towards strong enforcement as they impose greater penalties on tourism enterprises.

##### Proof

With other parameters unchanged, will make $y < \frac{{F_{1} - C_{1} - zW_{2} }}{{W_{1} + F_{1} - zV}}$, as $F_{1}$ increases, will eventually stabilize at $x = {1}$, i.e. the strategic behavior of the local government is chosen to be strongly enforced. On one hand, higher penalties imposed by local governments on tourism enterprises can lead to lower participation in tourism carbon offset, necessitating increased regulation, active guidance, and urging from local governments to encourage tourism enterprises to participate. On the other hand, the increased penalties can generate fiscal revenue for the local government, which can be used to cover some implementation costs or converted into carbon offset funds to incentivize other proactive stakeholders, such as providing subsidies for tourists, offering incentives for scientific research institutions, and so on.

##### Proposition 4

The greater the image gain for local governments, the more their behavioral strategy evolves towards a strong enforcement strategy.

##### Proof

With other parameters unchanged, will make $y < \frac{{F_{1} - C_{1} - zW_{2} }}{{W_{1} + F_{1} - zV}}$, as $V$ increases, will eventually stabilize at $x = {1}$, i.e. the strategic behavior of the local government is chosen to be strongly enforced. In general, with effective guidance and regulation from local governments, the probability of tourism enterprises responding positively to carbon offset policies will increase, leading to an increase in tourist participation. As a result, local governments will be able to successfully achieve the carbon peaking and carbon–neutral targets set by the central government, enhancing their credibility and reputation. This will also encourage local governments to adopt a strong implementation strategy for carbon offset policies.

#### Evolutionary game model for tourism enterprises

The expected benefits $E_{21}$ and $E_{22}$ of active and passive responses by tourism enterprises and the average benefits $\overline{{{\rm E}_{2} }}$ respectively are

$$ E_{21} = R_{3} - C_{3} + xW_{1} $$

$$ E_{22} = R_{2} - C_{2} - xF_{1} $$

$$ \overline{{{\text{E}}_{2} }} = {\text{y}}E_{21} + \left( {1 - y} \right)E_{22} $$

From this, the first-order derivatives of the replicator dynamics equations for tourism enterprises $F(y)$, $y$ can be obtained $\frac{dF(y)}{{dy}}$.

$$ F(y) = \frac{dy}{{dt}} = y(E_{21} - \overline{{E_{2} }} ) = y\left( {1 - y} \right)(C_{2} - C_{3} - R_{2} + R_{3} + xW_{1} + xF_{1} ) $$

$$ \frac{dF(y)}{{dy}} = (1 - 2y)(C_{2} - C_{3} - R_{2} + R_{3} + xW_{1} + xF_{1} ) $$

At the same time, making $G(x) = C_{2} - C_{3} - R_{2} + R_{3} + xW_{1} + xF_{1}$,

Since $\frac{dG(x)}{{dx}} = W_{1} + F_{1} > 0$, $G(x)$ are monotonically increasing functions, then $x = \frac{{C_{3} - C_{2} + R_{2} - R_{3} }}{{W_{1} + F_{1} }}$.

Let $x_{0} = x = \frac{{C_{3} - C_{2} + R_{2} - R_{3} }}{{W_{1} + F_{1} }}$, be discussed in three cases.

I. The When $x_{0} = x$, $G(x) = 0$, then $F(y) = 0$, $y$ take any number of stable states.
II. The When $$x_{0} > x$$$$G(x) > 0$$, then $$\frac{dF(y)}{{dy}}\left| {_{y = 0} } \right. < 0$$, $$\frac{dF(y)}{{dy}}\left| {_{y = 1} } \right. > 0$$, $$y = 0$$ are evolutionary stability points and the tourism enterprises ‘s strategic choice is to respond negatively.
III. The When $$x_{0} < x$$$$G(x) < 0$$, then $$\frac{dF(y)}{{dy}}\left| {_{y = 0} } \right. > 0$$, $$\frac{dF(y)}{{dy}}\left| {_{y = 1} } \right. < 0$$, $$y = 1$$ are evolutionary stability points and the tourism enterprises ‘s strategy choice is to respond positively.

Based on the above analysis, the following proposition is obtained.

##### Proposition 5

The greater the incentives offered by local governments to tourism enterprises, the more their strategic behavior evolves towards positive coping strategies.

##### Proof

With other parameters unchanged, will make ${\text{x}} = \frac{{C_{3} - C_{2} + R_{2} - R_{3} }}{{W_{1} + F_{1} }}$, as $W_{1}$ increases, will eventually stabilize at $y = {1}$, i.e. the strategic behavior of tourism enterprises choose to respond positively. The incentives provided by local governments to tourism enterprises can significantly reduce the costs associated with active response. Some tourism enterprises may even use these incentive funds to invest in technological transformation, upgrade their operations, or develop new products and projects. As a result, tourism enterprises may be more willing to adopt a proactive response strategy.

##### Proposition 6

The greater the penalties imposed by local governments on tourism enterprises, the more their strategic behavior evolves toward an active coping strategy.

##### Proof

With other parameters unchanged, will make $x < \frac{{C_{3} - C_{2} + R_{2} - R_{3} }}{{W_{1} + F_{1} }}$, as $F_{1}$ increases, will eventually stabilize at $y = {1}$, i.e. the strategic behavior of the tourism enterprises is chosen to respond positively. It is rational for tourism enterprises to choose a negative response strategy when their operating costs are low, their revenues are high, and their profits are substantial. However, when the local government increases penalties for non-compliance, the tourism enterprises ‘s profits will be reduced and its social image will be affected, which may prompt the operator to reconsider its response strategy.

##### Proposition 7

The smaller the difference between the operating profit of a tourism enterprises choosing a negative coping strategy and the operating profit of a positive coping strategy, the smaller the evolution of its strategic behavior towards a positive coping strategy.

##### Proof

With other parameters held constant, will make $x < \frac{{C_{3} - C_{2} + R_{2} - R_{3} }}{{W_{1} + F_{1} }}$, as the value of $C_{3} - C_{2} + R_{2} - R_{3}$ decreases, eventually, stabilize at $y = {1}$, i.e. the strategic behavior of the tourism operator chooses to respond positively. As the expected gains from carbon offset in the tourism industry increase, such as through technological transformation and upgrading, enhancing market competitiveness, and fostering a green corporate culture, tourism enterprises are more likely to adopt a positive coping strategy. However, if the benefits from traditional business practices are deemed to be greater, tourism enterprises may resort to negative coping strategies.

#### Evolutionary game model for tourists

The expected benefits $E_{31}$ and $E_{32}$ for tourists choosing to participate and not to participate, and the average benefits $\overline{{E_{3} }}$ respectively are:

$$ E_{31} = U_{1} - C_{4} + xW_{2} $$

$$ E_{32} = U_{2} - C_{5} $$

$$ \overline{{{\text{E}}_{3} }} = {\text{z}}E_{31} + \left( {1 - z} \right)E_{32} $$

From this, the first-order derivatives of the replicator dynamics equations for the tourist $F(z)$, $z$ can be obtained $\frac{dF(z)}{{dz}}$.

$$ F(z) = \frac{dz}{{dt}} = z(E_{31} - \overline{{E_{3} }} ) = z(1 - z)( - C_{4} + C_{5} + U_{1} - U_{2} + xW_{2} ) $$

$$ \frac{dF(z)}{{dz}} = (1 - 2z)( - C_{4} + C_{5} + U_{1} - U_{2} + xW_{2} ) $$

At the same time, making $I(x) = - C_{4} + C_{5} + U_{1} - U_{2} + xW_{2}$,

Since $\frac{dI(x)}{{dx}} = W_{2} > 0$,$I(x)$ are monotonically increasing functions, then $x = \frac{{C_{4} - C_{5} - U_{1} + U_{2} }}{{W_{2} }}$.

Let $x_{0} = x = \frac{{C_{4} - C_{5} - U_{1} + U_{2} }}{{W_{2} }}$, be discussed in three cases.

I. The $x_{0} = x$, $I(x) = 0$, then $F(z) = 0$, $z$ take any number, are stable states.
II. The When $$x_{0} > x$$$$I(x) > 0$$, then $$\frac{dF(z)}{{dz}}\left| {_{z = 0} } \right. < 0$$, $$\frac{dF(z)}{{dz}}\left| {_{z = 1} } \right. > 0$$, $$z = 0$$ are evolutionary stability points and the tourist’s strategy choice is non-participation.
III. The When $$x_{0} < x$$$$I(x) < 0$$, then $$\frac{dF(z)}{{dz}}\left| {_{z = 0} } \right. > 0$$, $$\frac{dF(z)}{{dz}}\left| {_{z = 1} } \right. < 0$$, $$z = 1$$ are evolutionary stability points and the tourist’s strategy choice is participation.

Based on the above analysis, the following proposition is obtained.

##### Proposition 8

The greater the incentives offered by local governments to tourists, the more their strategic behavior evolves toward a participation strategy.

##### Proof

Holding other parameters constant, will make $x < \frac{{C_{4} - C_{5} - U_{1} + U_{2} }}{{W_{2} }}$, as $W_{2}$ increases, eventually stabilize at $z = {1}$, i.e. the tourist’s strategic behavior choice is participation. Likewise, the opportunity cost for tourists to participate in carbon offset tends to increase. However, incentives offered by local governments can significantly reduce this cost, thereby motivating tourists to choose to participate in carbon offset.

##### Proposition 9

The smaller the difference between the utility of the tourist’s choice of the non-participation strategy and the utility of the choice of the participation strategy, the more his strategic behavior evolves towards the participation strategy.

##### Proof

With other parameters held constant, will make $x < \frac{{C_{4} - C_{5} - U_{1} + U_{2} }}{{W_{2} }}$, as the value of $\frac{{C_{4} - C_{5} - U_{1} + U_{2} }}{{W_{2} }}$ decreases, eventually stabilizing at $z = 1$, i.e. the tourist’s strategic behavior choice is participation. Through participating in carbon offset, tourists can obtain a greater sense of engagement and experience, as well as other benefits. However, if tourists do not participate in carbon offset, their behavior tends towards a non-participation strategy, as they can still achieve the same level of utility while reducing the cost of their tourism consumption.

### Tripartite evolutionary stability analysis

The above are the equilibrium conditions for each independent game agent to reach a stable strategy. To explore the equilibrium state of the final stable strategy under the joint effect of the three parties, the first method of Lyapunov is employed here to analyze the asymptotic stability of the tripartite replicator dynamics equations. This involves examining the distribution of the eigenvalues of the Jacobian matrix of the tripartite replicator dynamics equations to determine the stability of the system at a particular point.

Setting $F(x) = {0,}\,F(y) = {0,}\,F(z) = {0}$ reveals eight pure strategy Nash equilibrium points in the game process among local governments, tourism enterprises, and tourists, denoted as $E_{a} = (0,0,0)$, $E_{b} = (1,0,0)$, $E_{c} = (0,1,0)$, $E_{d} = (0,0,1)$, $E_{e} = (1,1,0)$, $E_{f} = (1,0,1)$, $E_{g} = (0,1,1)$ and $E_{h} = (1,1,1)$. By calculating the first partial derivatives of $F(x)$, $F(y)$, and $F(z)$, the Jacobian matrix can be obtained:

$$ J = \left[ {\begin{array}{*{20}c} {\frac{\partial F\left( x \right)}{{\partial x}}} & {\frac{\partial F\left( x \right)}{{\partial y}}} & {\frac{\partial F\left( x \right)}{{\partial z}}} \\ {\frac{\partial F\left( y \right)}{{\partial x}}} & {\frac{\partial F\left( y \right)}{{\partial y}}} & {\frac{\partial F\left( y \right)}{{\partial z}}} \\ {\frac{\partial F\left( z \right)}{{\partial x}}} & {\frac{\partial F\left( z \right)}{{\partial y}}} & {\frac{\partial F\left( z \right)}{{\partial z}}} \\ \end{array} } \right] $$

To analyze the stability of the equilibrium points in the evolutionary game of tourism carbon offset, we substitute the eight pure strategy Nash equilibrium points into the Jacobi matrix and obtain the eigenvalues. which are shown in Table 2.

**Table 2:** Eigenvalues of Jacobi matrix at each equilibrium point.

Balancing point	Eigenvalue λ_1_	Eigenvalue λ_2_	Eigenvalue λ_3_
(0, 0, 0)	F_1_−C_1_	C_2_−C_3_−R_2_ + R_3_	C_5_−C_4_ + U_1_−U_2_
(1, 0, 0)	C_1_−F_1_	C_2_−C_3_ + F_1_−R_2_ + R_3_ + W_1_	C_5_−C_4_ + U_1_−U_2_ + W_2_
(0, 1, 0)	−C_1_−W_1_	C_3_−C_2_ + R_2_−R_3_	C_5_−C_4_ + U_1_−U_2_
(0, 0, 1)	F_1_−C_1_−W_2_	C_2_−C_3_−R_2_ + R_3_	C_4_−C_5_−U_1_ + U_2_
(1, 1, 0)	C_1_ + W_1_	C_3_−C_2_−F_1_ + R_2_−R_3_−W_1_	C_5_−C_4_ + U_1_−U_2_ + W_2_
(1, 0, 1)	C_1_−F_1_ + W_2_	C_2_−C_3_ + F_1_−R_2_ + R_3_ + W_1_	C_4_−C_5_−U_1_ + U_2_−W_2_
(0, 1, 1)	V−C_1_−W_1_−W_2_	C_3_−C_2_ + R_2_−R_3_	C_4_−C_5_−U_1_ + U_2_
(1, 1, 1)	C_1_ -V + W_1_ + W_2_	C_3_−C_2_−F_1_ + R_2_−R_3_−W_1_	C_4_−C_5_−U_1_ + U_2_−W_2_

Based on the Lyapunov discriminant, an equilibrium point is asymptotically stable if all eigenvalues of the Jacobi matrix are negative. If all eigenvalues are positive, the equilibrium point is unstable, and if there are one or two positive eigenvalues, the equilibrium point is a saddle point^59^. According to the matrix eigenvalues of each equilibrium point in Table 2, the eigenvalues of the equilibrium points $E_{a} (0,0,0)$ and $E_{h} (1,1,1)$ are both negative, as shown in Table 3, so $E_{a} (0,0,0)$ and $E_{h} (1,1,1)$ are stable points. The two scenarios are discussed below:

**Table 3:** Stability determination of equilibrium points in the evolutionary game of tourism carbon offset.

Balancing point	$\gamma_{1}$	$\gamma_{2}$	$\gamma_{3}$	Stability	Conditions
*E*_a_(0, 0, 0)	–	–	–	Stabilisation points	*F*_1_ < *C*_1_; *R*_3_–*C*_3_ < *R*_2_–*C*_2_; *U*_1_–*C*_4_ < *U*_2_–*C*_5_
*E*_b_ (1, 0, 0)	+	Uncertainty	Uncertainty	Instability points or saddle points	
*E*_c_ (0, 1, 0)	–	+	–	Saddle point	
*E*_d_ (0, 0, 1)	Uncertainty	+	+	Unstable or saddle point	
*E*_e_ (1, 1, 0)	+	–	+	Saddle point	
*E*_f_ (1, 0, 1)	+	+	–	Saddle point	
*E*_g_(0, 1, 1)	+	–	–	Saddle point	
*E*_h_ (1, 1, 1)	–	–	–	Stabilisation points	*C*_1_ + *W*_1_ + *W*_2_ < *V*; –*C*_2_–*F*_1_ + *R*_2_ < –*C*_3_ + *R*_3_ + *W*_1_; –*C*_5_ + *U*_2_ < *U*_1_ + *W*_2_–*C*_4_

In Scenario 1, when $F_{1} < C_{1} , R_{3} - C_{3} < R_{2} - C_{2}$ and $U_{1} - C_{4} < U_{2} - C_{5}$, the tripartite evolution stable strategy is (0, 0, 0), which corresponds to weak enforcement, negative response, and non-participation. In the short term, local governments will opt for a weak enforcement strategy if the costs of strong enforcement exceed the penalties imposed on tourism enterprises. Tourism enterprises will choose a negative response strategy if the net profit from active response is less than that from the negative response. Additionally, tourism enterprises will choose not to participate if the net utility from tourism enterprises participation is less than the net utility from non-participation.

In Scenario 2, If $- C_{2} - F_{1} + R_{2} < R_{3} - C_{3} + W_{1}$ and $U_{2} - C_{5} < U_{1} - C_{4} + W_{2}$, the evolutionary stabilization strategy for all tripartite is (1, 1, 1), meaning they will choose to implement strong enforcement, active response, and participation in carbon offset. In the long run, local governments will choose the strong enforcement strategy when the perceived image gain is greater than the sum of the operating costs and the financial expenditure of the incentive. Tourism enterprises will choose the active response strategy when the local government incentive funds are sufficient to cover the net profit loss resulting from choosing to be active in carbon offset. If the local government incentive funding is higher than the net utility loss caused by tourists participating in carbon offset, tourists will choose the participation strategy.

## Simulation analysis of the tripartite evolutionary game

Numerical simulation can verify the feasibility and accuracy of the conclusions of the game, while reflecting the evolution of the game of each subject under different parameter conditions, and intuitively reflecting the size of the trend of the influence between the variables^60^. Therefore, MatlabR2021a software is used to numerically simulate the model to more intuitively analyze the impact of the strength of local government incentives and constraints on the behavioral decisions of other subjects. We take the optimal steady state situation *E*_*h*_ (1, 1, 1) as the basis, consider the interaction between the parameters, and assign values to the model parameters. In terms of setting the values of the parameters related to the limited rationality factor, referring to the research of Van^61^ and Liu^49^, the limited rationality individual’s pursuit of risk in the face of loss is stronger and more sensitive than gain, and the cost of irresponsible environmental behavior is much lower than the cost of responsible environmental behavior^62^, so the coefficients are set to C1 = 0.65, C2 = 0.4, C3 = 0.7, C4 = 0.55, and C5 = 0.2. The subject’s responsible environmental behavior is continuously affected by the policy, so the subject’s responsible environmental behavior is more sensitive than gain, and the cost of responsible behavior is much lower than cost of responsible behavior. According to Wang^63^ and Senbil’s^64^ research, in order to take into account social stability and economic development, local governments usually do not punish enterprises for irresponsible environmental behavior too much, and due to the constraints of regulatory costs, they do not punish the public for irresponsible environmental behavior. The probability of punishing the public for environmentally irresponsible behavior is also low due to the constraints of regulatory costs. The value of incentives for the public as a group is also lower than that for individuals with clear goals. Based on the relevant literature and the obtained stabilization conditions, the parameter values for the decision influence coefficients are set as follows: W1 = 0.6; W2 = 0.45; F1 = 0.6.

### Evolutionary path of the tripartite game

**Figure 1:**
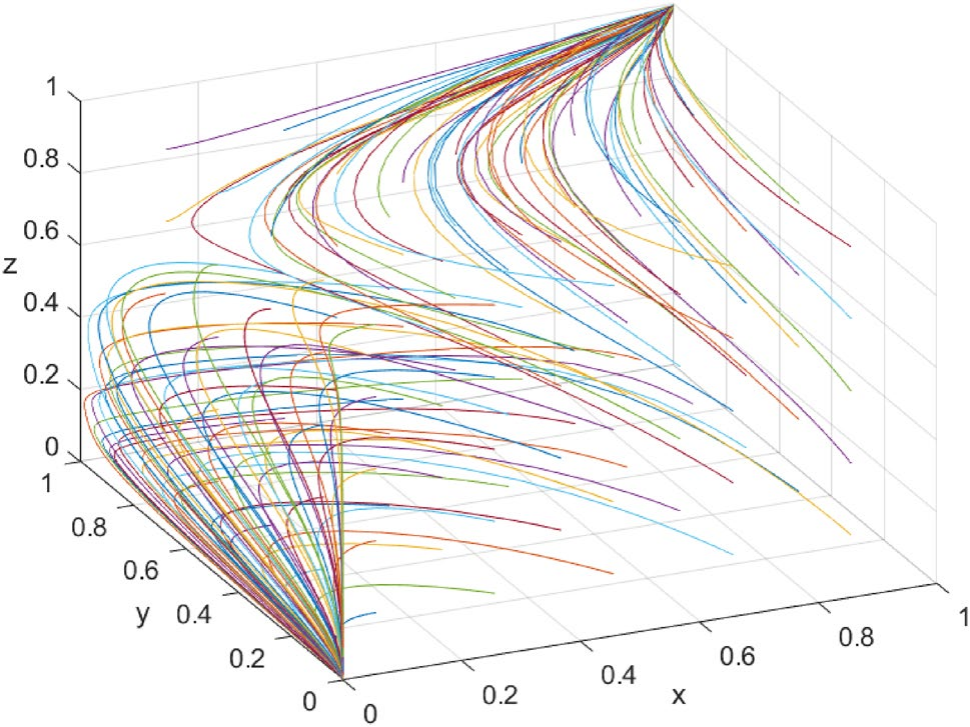
System evolution path diagram.

By substituting the arrays into the model, the simulation results presented in Fig. 1 reveal that the evolutionarily stable state of the system is (1, 1, 1), which aligns with the conclusion of scenario 2. The simulation analysis confirms the stability of the subject’s strategies, thereby validating the model. To avoid the impact of the initial strategies of the parties on the evolution of the system and to facilitate comparison of the evolution of the tripartite strategy ratio, the initial probabilities of the local government, tourism enterprises, and tourists are assumed to be 0.5. Building on this, the influence of local government constraints (*F*_*1*_) and incentives (W_1_ and W_2_) on the evolutionary strategy of the game’s subject are analyzed.

### Influence of constraint strength on the evolutionary outcome of the system

**Figure 2:**
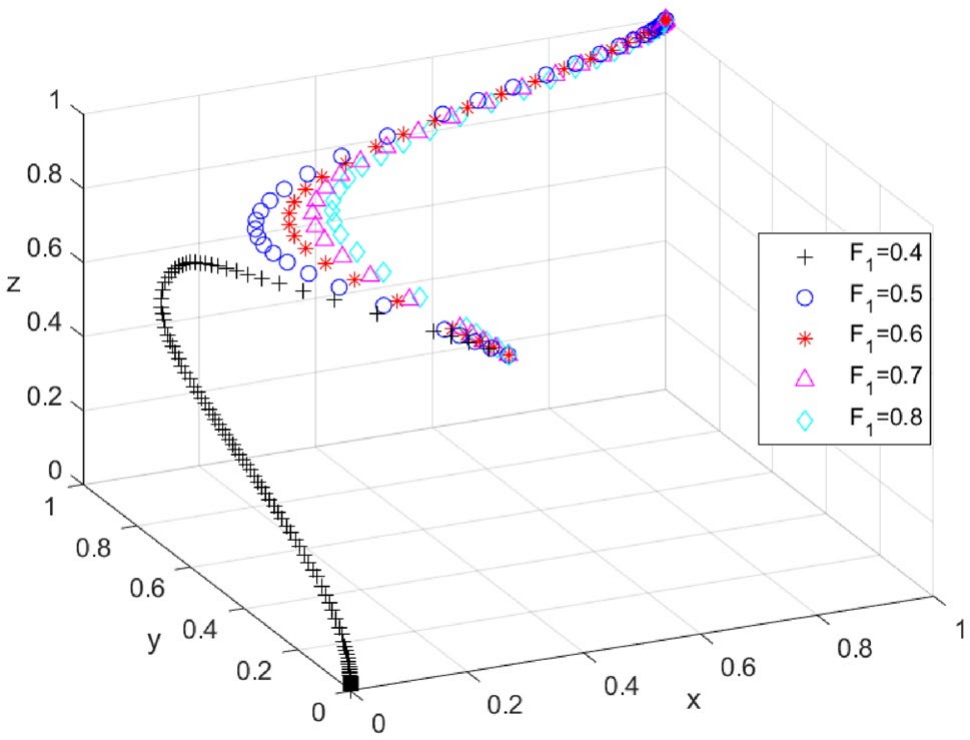
Effect of changes in *F*_*1*_ on the evolutionary outcome of the system.

To investigate the influence of variations in the enforcement intensity of local governments on the outcomes of system evolution, we assume the values of* F*_*1*_ are set at 0.4, 0.5, 0.6, 0.7, and 0.8, respectively. The simulation results are presented in Fig. 2. As the intensity of local government penalties increases, the evolutionary paths of the three parties shift from (0, 0, 0) to (1, 1, 1), with the critical value of *F*_*1*_ ranging between 0.4 and 0.5. When the penalty intensity is below this critical threshold, x, y, and z converge to 0. At this point, local governments opt for a weak enforcement strategy, tourism enterprises adopt a passive response strategy, and tourists choose a non-participation strategy. When the penalty intensity exceeds this critical value, x, y, and z converge to 1. At this juncture, local governments adopt a strong enforcement strategy, tourism enterprises engage in proactive response strategies, and tourists opt to participate. The results of the evolution indicate that the constraint mechanism is a crucial factor in altering the evolutionary path of the system. The system will evolve towards (1, 1, 1) only when the penalty intensity exceeds a certain critical value.

### Effect of incentive intensity on the evolutionary outcome of the system

**Figure 3:**
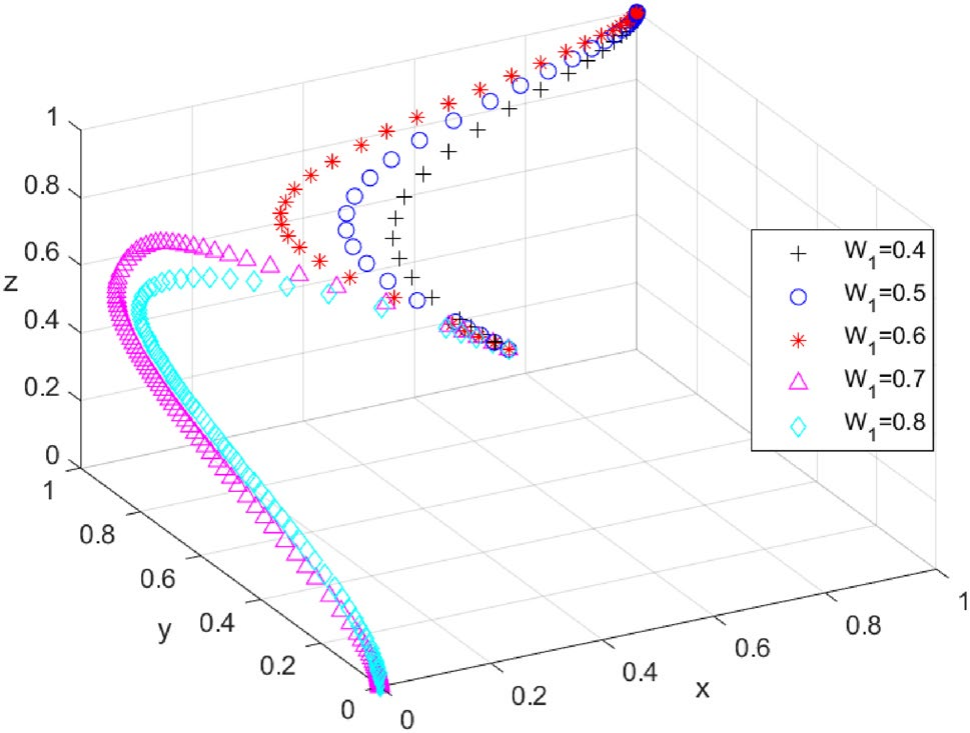
Effect of changes in W1 on the evolutionary outcome of the system.

To investigate the impact of local government incentives on tourism enterprises on the system’s evolutionary results, different values of W_1_ are assumed, ranging from 0.4 to 0.8, and the simulation results are presented in Fig. 3. When the incentive is below 0.6, the reward system promotes the active participation of local government, tourism enterprises, and tourists in carbon offset activities, and the evolutionary path of the tripartite progresses from (1, 1, 1) to (0, 0, 0) as the incentive of the local government increases. The critical value of W_1_ is found to be between 0.6 and 0.7, and when the incentive is higher than this critical value, x, y, and z converge to 0. When the local government’s incentive to tourism enterprises becomes too high, it can further increase the financial burden on the local government, and in the absence of incentives, tourism enterprises and tourists may be reluctant to participate. When the incentives are below this threshold, x, y, and z converge to 1, indicating that the local government chooses a strong enforcement strategy, tourism enterprises opt for an active response strategy, and tourists choose a participation strategy. These evolutionary results highlight the importance of the incentive mechanism of the local government towards tourism enterprises in changing the system’s evolutionary path. Therefore, controlling the incentive strength at a certain critical value is crucial for the system to evolve towards (1, 1, 1).

**Figure 4:**
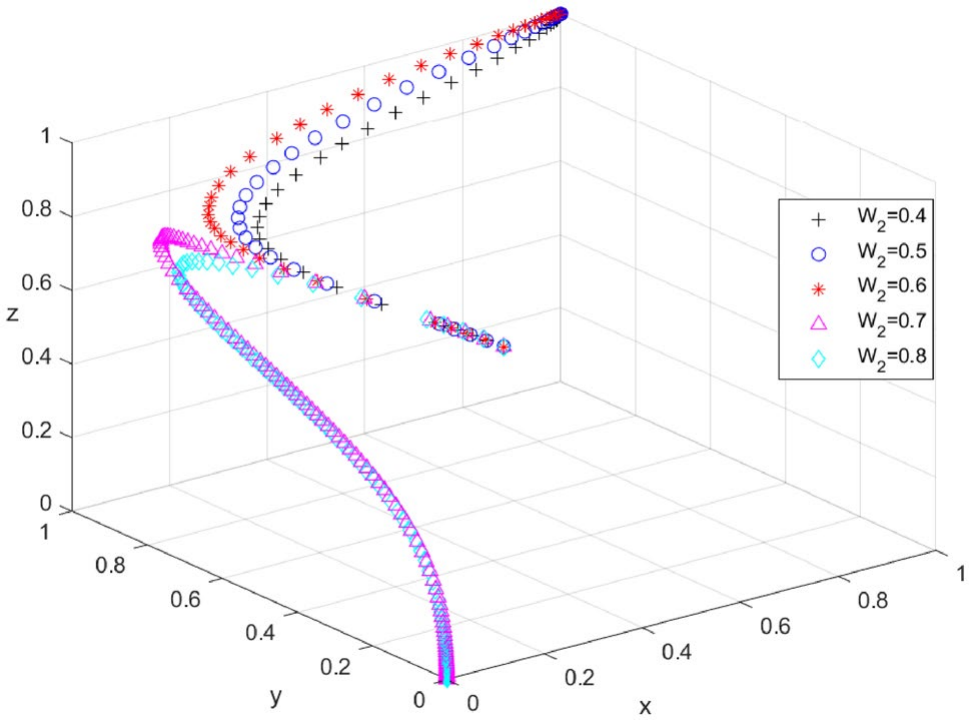
Effect of W2 changes on system evolutionary outcomes.

To investigate the influence of changes in the intensity of local government rewards for tourists on the evolutionary outcomes of the system, we assumed values of W_2_ to be 0.4, 0.5, 0.6, 0.7, and 0.8, respectively. The simulation results are presented in Fig. 4. As the local government incentives increase, the evolutionary path of the tripartite transitions from (1, 1, 1) to (0, 0, 0), with the critical value of W_2_ ranging between 0.6 and 0.7. When the reward exceeds this critical value, x, y, and z converge to 0, indicating that excessive local government rewards for tourists could also increase the financial burden on the local government. At this point, the local government is likely to choose a weak enforcement strategy, while tourists may be reluctant to participate in carbon offset. Consequently, tourism enterprises may adopt a negative coping strategy. On the other hand, when the incentive is below this threshold, x, y, and z converge to one. At this point, the local government adopts a strong enforcement strategy, the tourism enterprises chooses a positive coping strategy, and the tourist participates actively. The evolutionary results demonstrate that the local government’s incentive mechanism for tourists is a crucial factor that shapes the evolutionary path of the system, and the system will only evolve when the incentive strength is controlled at a certain critical value.

## Conclusions and discussion

This paper obtains the following conclusions by constructing a game model of local government, tourism operators and tourists’ tripartite participation in the evolution of carbon offsetting, and analyzing the effective interval of incentive and constraint mechanisms through simulation:

1. In the tourism carbon offset game relationship, local governments, tourism enterprises and tourists are core stakeholders and their strategic decisions are interdependent. When the local government’s incentives for tourism enterprises and tourists are larger, the implementation cost is higher, and the local government’s carbon offset strategy evolves toward weak implementation; when the penalty for tourism enterprises is larger and the image gain is obtained, the local government evolves toward strong implementation. Strong constraints and weak incentives of local governments are effective carbon offsetting means.
2. The constraint and incentive mechanisms have different effects on the evolution of the stabilization strategy of the whole system. Under the weak constraint mechanism, i.e., when the penalty is lower than 0.5, the tripartite show negative participation; as the local government’s penalty increases, the faster the system evolves to the (1, 1, 1) stabilization strategy, the more the tripartite ‘ participation increases, and the critical value of the penalty is between 0.4 and 0.5.
3. The critical point of the local government’s reward for enterprises and tourists is between 0.6 and 0.7, and when the reward is below this level or less, the tripartite will actively participate in carbon offsetting. The faster the system evolves to a (0, 0, 0) stable strategy when the reward is stronger, i.e., under strong incentives, as the reward continues to increase, the local government will choose a weak implementation strategy due to the financial burden, which in turn leads to the negative participation of tourism enterprises and tourists as well. Therefore, in the implementation process of tourism carbon offsetting, scientific design of constraint and incentive mechanism can effectively improve the enthusiasm of local governments, tourism operators and tourists.

Ecological compensation plays an important role in regulating the relationship between human activities and the ecological environment^65,66^. Similarly, it is feasible to regulate the relationship between tourism development and climate change through carbon offsets. Stakeholders such as the government, tourism enterprises and tourists play different roles in tourism carbon offsetting, and their differentiated interest objectives lead to different strategic choices, so the phenomenon of collective action dilemma of tourism carbon offsetting is inevitable. As for the collective action dilemma in the governance of public affairs such as environmental pollution control, biodiversity protection and natural resources utilization, studies by Zhou^67^ and Yuling ^68^ show that the intervention of government incentive and constraint regulation can effectively regulate the behavioral strategies of stakeholders to evolve in a positive direction. Due to the externality of carbon offsetting behavior, without government incentives and constraints regulation, the increase of additional costs in the early stage of the implementation of tourism carbon offsetting is unavoidable, which will lead to the lack of active participation of tourism enterprises and tourists, i.e., energy saving and emission reduction in the market failure^69^. The intervention of government incentives and constraints regulation will have a positive effect on the stability of the tourism carbon offsetting system, which is conducive to increasing the positive participation of tourism enterprises and tourists. However, there is a maximum threshold for incentive and a minimum threshold for constraint. This conclusion can cause us to think that the government, as a representative of public interest and an implementer of environmental protection work, should increase the responsibility of tourism enterprises and tourists in the carbon offsetting process through the policy tool of combining rewards and punishments, but it should avoid too much administrative intervention^70^, and the short-sighted effect of measuring the effectiveness of the implementation of the policy tool by economic benefits only, and should dynamically adjust the policy of penalties and incentives to synergistically push forward the low-carbon transformation and ecological and environmental benefits of the tourism industry. The penalty and incentive policies should be dynamically adjusted to synergistically promote the low-carbon transformation of the tourism industry and the improvement of ecological and environmental benefits.

Based on the findings of the above study, the following management insights are suggested:

Firstly, a penalty mechanism for non-compliance should be established. Penalties for tourism enterprises’ negative response to carbon offsets should be increased, and the amount of fines imposed on tourism enterprises should be higher than the additional revenue gained from their negative response, thus raising the cost of negative response by tourism enterprises. Information on penalties imposed on non-compliant tourism enterprises should be disseminated through news media or official government websites on information sharing platforms for public display, increasing exposure of non-compliance to create a social deterrent effect, and incorporating penalty information into the industry credit system. Creating a healthy and orderly industry development environment will help improve the decision-making behavior of tourism enterprises and promote the active implementation of carbon offset policies.

Secondly, an incentive support mechanism should be established. An important factor in the low level of low-carbon development in China’s tourism industry is the lack of innovation capacity. Throughout the process of project innovation and participation, tourism enterprises’ operating costs and tourists’ consumption costs will further increase. As new projects are characterized by high investment and low benefits in the initial application stage, it is difficult to achieve scale in project innovation, and coupled with the instability of the tourism market, tourism enterprises have little profit margin or even loss, tourists’ experience is not strong and consumption is not cost-effective. Therefore, the local government should improve the incentive support mechanism according to its financial capacity, adopt a flexible incentive approach, and set a reasonable incentive amount to reduce the operating costs of tourism enterprises and the consumption costs of tourists.

Thirdly, a low-carbon development propaganda and education mechanism should be established. China’s targets for reaching carbon peak and carbon neutrality were set recently and have limited coverage, resulting in insufficient public awareness and understanding of these concepts. Carbon peaking and carbon neutrality should be integrated into the training systems for ecological civilization education of staff at all levels of government departments, achieving institutionalization of government-led low-carbon education. Carbon neutrality should also be incorporated into the daily operation and safety management system of enterprises, making low-carbon education for enterprises a daily practice. Carbon neutrality should be incorporated into the national education system to standardize low-carbon education for schools and the community management system, making low-carbon education for the public a regular practice. Integrate carbon peaking and carbon neutrality into community management systems to normalize public education on low-carbon practices.

Tourism carbon offsetting is an important means to address tourism carbon emissions, involving multiple stakeholders such as government (central government, local government), tourism enterprises, community residents, tourists, news media, financial institutions and research institutes. This paper focuses on the evolutionary game among local governments, tourism enterprises and tourists. The research team is constrained by the systematic complexity and technical difficulty of the four-way evolutionary game, and the article has certain limitations in the selection of interest subjects and does not consider the impact of the top-level design of the central government on tourism carbon offset. The central government is the commissioner and guide of tourism carbon offset policy, with the functions of providing institutional guarantee, financial support, carbon trading market and platform building. At the same time, it has strong financial, administrative and legal resources, with a strong ability to draw resources, social integration and policy implementation. The strategy intensity and type of strategy of the central government will also directly affect the behavior of other stakeholders. In the future, the central government, local governments, tourism enterprises and tourists will be simultaneously included in the evolutionary game model to explore the influence of the central government in carbon offsetting on the carbon offsetting decisions of other stakeholders through administrative means, legal measures and industrial policies.

## Data Availability

The datasets used and analyzed during the current study are available from the first author (Q.L.) upon reasonable request. Data used in the present study is publicly available at https://tieba.baidu.com/p/8906794432?pid=149826017281&cid=0#149826017281.
